# Height loss caused by bent posture: A risk factor for stroke from ENT clinic - is it time to reconsider the physical examination?

**DOI:** 10.3109/00016489.2011.587451

**Published:** 2011-06-01

**Authors:** Koichi Tsunoda, Research Group on The Relationship of Bent Posture and Stroke, National Hospital Organization (NHO)

**Affiliations:** National Hospital Organization (NHO), Tokyo, Japan

**Keywords:** Pharyngeal study, aging, oral examination, diagnosis, aberration, carotid artery

## Abstract

*Conclusion*. When excessive height loss occurs in the elderly, which may be indicated by a cervical-bent posture, examination of the head and neck should be performed to detect common carotid artery (CCA) and internal carotid artery (ICA) aberration. In such cases brain magnetic resonance imaging (MRI) examination should be conducted to determine whether infarction is present. *Objectives*. To examine the relationships among bent posture, height loss, aberration of carotid arteries, and ischemic stroke risk with a case-control study. *Methods:* Controls (*n* = 163) were selected from among patients who had undergone MRI of the brain because of otolaryngologic symptoms. Case patients (*n* = 72) were selected from among those whose primary diagnosis was cerebral infarction in the area served by the ICA. Both groups were age-matched between 65 and 84 years old. The neck and pharyngeal cavity in each of the 235 patients were examined to determine whether the carotid arteries exhibited aberration. Patients' current height and greatest lifetime height were recorded, along with presence or absence of bent posture and other stroke risk factors. *Results:* Height loss alone could predict stroke risk in 79.1% of patients: 90.2% based on carotid artery aberration and 91.4% when all risk factors (aberration of carotid artery, height loss, bent posture) were analyzed.

## Introduction

Advances in medical science have overcome many previously fatal diseases and contributed to increased longevity. The identification of risk factors for specific conditions has aided in their prevention while decreasing health-care expenditure. Continued research on identifying the risk factors for and causes of stroke and increase of therapeutic interventions for those identified as ‘at risk’ have already resulted in lower stroke morbidity and mortality rates in developed countries, but there is a need for further research.

Known risk factors for stroke include arrhythmia in the form of atrial fibrillation [1-3], diabetes [4,5], and heart valve anomalies [1,6]. About 80% of all strokes are of ischemic origin [7]. We previously suggested that aberrations of the carotid arteries in the head (internal carotid artery, ICA) [8] and neck (common carotid artery, CCA) [9], which may occur when the neck is bent forward, should be considered as new risk factors for ischemic stroke. These anomalies occur because of decrease in the distance that the carotid artery traverses between its central end and the skull base. Although a vein can contract in length and widen to adjust to this shorter distance, an artery cannot do so if it is atherosclerotic. Since it cannot shorten, the ICA is forced to bend. The ICA is located within the parapharyngeal space, the only nonrigid borders of which are the medial and inferior areas. For these reasons, the mouth is anatomically the most likely site for aberration of the ICA to occur. The CCA may also divert from its normal course. Marked aberration in patients with severe atherosclerosis causes turbulent blood flow that produces plaque and leads to brain infarctions.

The bent posture that occurs with aging is generally associated with changes in the spine, which result in height loss. While we initially focused on bent posture as a risk factor for stroke, evaluation of ‘bent posture’ in the physician's office is somewhat subjective. It would be worthwhile to consider whether height loss itself, which provides an easily available quantitative measure, could serve as a predictor.

A recent study reported that marked height loss (≥3 cm) in older men is independently associated with an increased risk of all-cause mortality and coronary heart disease [10]. This prospective study examined the effect of height loss over a 20-year period (starting in middle age) on subsequent overall mortality and the incidence of coronary heart disease and stroke in men. The height of 4213 men was measured between the ages of 40 and 59 years and again 20 years later between the ages of 60 and 79 years. Although height loss was not found to associate directly with stroke in the population studied, this relationship is clearly worthy of further investigation, especially in light of our reports on the effects of bent posture.

The aim of this study, therefore, was to examine the relationships among bent posture, height loss, and stroke risk based on our hypothesis. To compare the incidence of carotid artery aberration, a case-control study was performed in patients with brain infarction (case group) and those without brain infarction (control group) from a population that was referred to the ENT clinic.

## Material and methods

This case-control study was performed at the National Hospital Organization Tokyo Medical Center and 14 related hospitals after the Ethics Committee of each had approved the study protocol. Patients were selected based on the protocol criteria and all gave written informed consent before enrollment in this study between January 2005 and December 2008.

All cases and controls were 65-84 years of age to meet the protocol criteria. Controls were selected from among patients who had undergone magnetic resonance imaging (MRI) of the brain because they had experienced dizziness, hearing problems, or other otolaryngologic symptoms. Patients found to have experienced cerebellar hemorrhage or to have tumors based on MRI examination were excluded from the case group. Case patients were selected from among those whose primary diagnosis was cerebral infarction in the area of the ICA. Those with lacunar infarction were excluded from the case group. Patients with established risk factors for stroke, such as a history of atrial fibrillation, arrhythmia, heart valve disease, diabetes, blood clotting abnormalities (hematocrit >55%, platelet count >500 000/μl), and who were receiving anticoagulant therapy were excluded from the study. The remaining patients, in whom infarction was not identified, were assigned to the control group. In total, 235 patients (case group 72, control group 163) were selected for detailed examination. We examined the neck and pharyngeal cavity in each of these 235 patients in the seated position to determine whether the ICA and CCA exhibited aberration. Before the detailed examinations, workshops were held for physicians performing the study to ensure that all facilities used a standardized method. The physicians were instructed to conduct the examinations while the patients maintained a comfortable, stable seated posture. Patients' necks were palpated along both sternomastoid muscles (SCMs) to determine the presence of aberrations of the CCA. If the CCA was not parallel with the SCM but located anterior or posterior to the SCM with bruit, aberration of the CCA was diagnosed. If pharyngeal or nasopharyngeal fiberscopic examination found that the lateral pharyngeal wall pulsated and was deviated by the force of the ICA in the median direction (toward the inside of the oral cavity), the diagnosis was aberration of the ICA. We also recorded all patients' height (greatest lifetime height and current height), brain MRI findings, gender, age, blood pressure, cholesterol level, body weight, and smoking and alcohol consumption habits.

### Statistical analysis

For clarity, observational data (neck anteflexion and ICA/CCA aberration) were categorized as binary variables. Height loss was also categorized as a binary variable based on whether the difference in current height from the greatest lifetime height was ≥3 cm. First, the binary variables were summarized into a single case-control contingency table. Case-control contingency tables were also constructed separately for the male and female patient groups. The basic statistics of laboratory test values, means, and SDs were calculated by group and gender for each numerical variable of age, clinical data, and history of smoking status and alcoholic beverage consumption. To determine case and control differences, contingency table analysis using the Mantel-Haenszel procedure was adopted for each of the two binary variable tables. Among the significant binary variables, mutual odds ratios (ORs) with 95% confidence intervals (CIs) as well as case-control ORs were generated. For the numerical variables, two-way (case-control x gender) factorial analysis of variance (ANOVA) was conducted.

Through the primary analysis, variables with case-control sensitivity were screened as candidate risk factors. To take into account mutual risk, adjusted ORs with 95% CIs of each candidate factor were calculated by logistic regression modeling and their significance was assessed in the likelihood ratio test. The overall corrected predictions were examined in the final model.

Further analysis from the viewpoint of laterality was performed on the relationship between ICA/CCA aberration and brain infarction. Before the analysis, the factor of ICA/CCA aberration was classified into four categories (left side alone, both sides, right side alone, and none) based on the site of aberration, and the factor of brain infarction was classified into three categories (left hemisphere, bilateral hemispheres, and right hemisphere) based on the hemisphere in which infarction occurred. The association between the factors was examined in a 4 x 3 contingency table using the chi-square test to determine independence. To express laterality for each ICA/CCA category, the following laterality index (LI) was introduced: LI = ([left brain infarction alone] - [right brain infarction alone])/[subtotal in all three infarct categories]. Positive values indicate left-sided lateralization, whereas negative values indicate right-sided lateralization. All analyses were done using SPSS software (v. 17).

## Results

**Figure 1:**
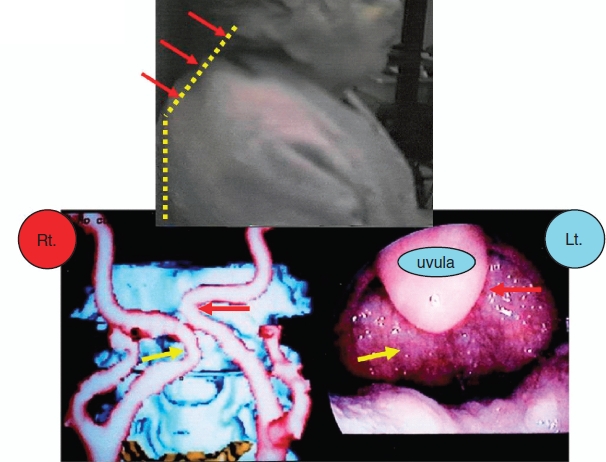
Top: actual bent-posture in an 84-year-old female. (yellow dots). Lower: three-dimensional computed tomography (CT) showing aberration of the internal carotid artery on both sides. Aberrant carotid arteries in the mouth. A submucosal swelling pulsates in synchrony with the patient's heartbeat. Aberration of the internal carotid artery. Reproduced with permission from Tsunoda K et al. Ann Intern Med 2003; 139:W-56 [8].

The intergroup differences between case and control patients were determined in the primary analysis. In this case-control study, 76.4% (55/72) of the case group showed severe height loss (≥3 cm); the values were 19.6% (32/163) in the control group. In the height loss group, 69.3% (61/88) of patients had aberrations of the ICA. Furthermore, 90.2% (55/61) of such patients belonged to the case (72 cases) group, which was revealed to include 63 (87.5%) patients with ICA aberration. Figure 1 illustrates examples of these findings.

In terms of demographic factors, the subjects were age-matched, but gender was unmatched as there were relatively fewer males in the control group. There were gender-specific case-control differences in all categorical factors including data on neck anteflexion (male, female, and total ORs with 95% CIs: 31.88 [9.09-111.75], 50.63 [16.31-157.15], and 44.04 [19.61-98.89], respectively) as well as nonin-vasive data on height loss (15.65 [5.22-46.93], 20.18 [7.13-57.14], and 13.24 [6.80-25.81], respectively) and ICA/CCA aberration (33.75 [7.85-145.10], 103.13 [26.38-403.21], and 88.08 [35.35-219.46], respectively). ICA/CCA aberration, which had the highest OR score among categorical factors, was also associated with neck anteflexion (male, female, and total ORs with 95% CI: 48.21 [12.92-179.91], 52.80 [17.81-156.52], and 51.18 [22.14-118.29], respectively), and height loss (58.50 [15.18-225.42], 20.54 [7.69-54.85], and 25.42 [12.08-53.50], respectively). Among numerical factors, diastolic/systolic blood pressure and high-density lipoprotein (HDL) cholesterol levels showed case-control differences, and only HDL cholesterol level was gender dependent (male/female mean with SD: 54.3 ± 17.1/63.1 ± 16.6) but no interaction effect between group and gender was found.

The principal results of logistic regression analysis of the significant noninvasive risk factors are listed in Table I. Neither diastolic nor systolic blood pressure was a significant potential risk factor for stroke. Differences in HDL cholesterol level between case and control patients also did not reach a statistically significant level, while the factors of height loss, neck anteflexion, and ICA/CCA aberration were all highly significant. The adjusted OR (and its 95% CI) for neck anteflexion was slightly lower than that for height loss.

**Table I:** Risk for ischemic stroke without established factors.

Variable	Adjusted ORs (95% CI)	*p* value
Categorical risk factors		
Gender	0.25 (0.09-0.75)	<0.05
Neck anteflexion	16.20 (2.54-103.33)	<0.001
Height loss (≥3 cm)	19.49 (3.07-123.48)	<0.001
Abnormal head/neck	23.39 (4.78-114.44)	<0.001
carotid arteries		
Numerical risk factors		
Diastolic blood pressure	0.99 (0.94-1.05)	NS
Systolic blood pressure	1.02 (0.99-1.05)	NS
HDL-cholesterol level	0.98 (0.95-1.01)	NS

**Figure 2:**
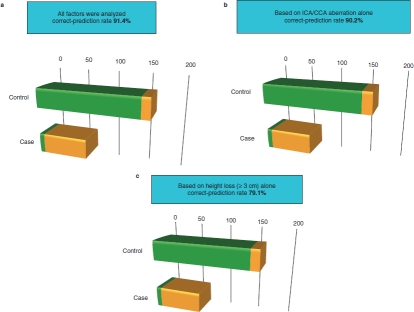
Stroke prediction based on possible risk factors. (a) Rates of discriminated stroke (orange) and normal (without stroke) (green) in control (upper) and case (lower) patients using all significant risk factors. (b) Based on ICA/CCA aberration alone. (c) Based on height loss (≥3 cm) alone.

Figure 2 illustrates the results of stroke prediction in the study population based on possible risk factors. The correct-prediction rate was 91.4% when all factors were analyzed (Figure 2a), which remained almost the same (90.2%) when ICA/CCA aberration alone was analyzed (Figure 2b). False-positive rates were 6.75% and 8.60%, respectively. Height loss alone correctly predicted stroke risk in 79.1% of patients (Figure 2c), with a false-positive rate of 19.6%.

The contingency table relating the occurence and laterality of ICA/CCA aberration to laterality of brain infarction is shown in Table II. The chi-square test confirmed the dependency of stroke laterality on the site of ICA/CCA aberration (*p* < 0.001). There were nearly twice as many patients with ICA/CCA aberration on the right side alone compared with those with aberration on the left side alone. The overall laterality of brain infarction also had a tendency for right hemisphere dominance (LI = -0.15). However, such dominance was determined exclusively by the subgroup with carotid artery aberration on the right side alone (LI = -0.85).

**Table II:** Laterality in association between site of aberration of head/neck carotid arteries and brain infarction.

	Hemisphere of brain infarction				
	
Laterality	Left (%)	Bilateral (%)	Right (%)	Subtotal (%)	LI*
Left side alone	5 (6.9)	4 (5.6)	2 (2.8)	11 (15.3)	+0.27
Both sides	7 (9.7)	18 (25.0)	7 (9.7)	32 (44.4)	0
Right side alone	0 (0.0)	3 (4.2)	17 (23.6)	20 (27.8)	-0.85
None	5 (6.9)	2 (2.8)	2 (2.8)	9 (12.5)	+0.33
Total	17 (23.5)	27 (37.6)	28 (38.9)	72 (100)	-0.15

* LI (laterality index) = ([left brain infarction] - [right brain infarction])/[subtotal].

## Discussion

The results of this study strongly support our previous reports relating bent posture to aberration of the carotid arteries and, therefore, to risk of stroke [8,9]. In our study population, which was selected for stroke-like symptoms, carotid artery aberration was the best predictor of stroke in the test group while still producing few false-positive results in the control group. Our results further extend those of Wannamethee et al. [10], in demonstrating the power of height loss ≥3 cm as a predictor of health risk. While height loss failed as a predictor of stroke in the general elderly male population in that study, it proved to be a better predictor than the occurrence of bent posture for our population. We found that patients with height loss generally had bent posture, which included not only cervical bending (kyphosis) but also some thoracic bending. Interestingly, the incidence of stroke was significantly higher in patients with cervical-bent compared with thoracic-bent posture. We speculate that this was because the diameters of other arteries were greater than those of the cervical arteries.

In conclusion, we strongly suggest that it is time to reconsider the current physical diagnostic procedures in patients aged 65 years and older. When height loss of 3 cm or more occurs in the elderly, possibly indicated by cervical-bent posture, examinations of the head, neck, and parapharyngeal cavity with simultaneous palpation of the bilateral SCM should be performed to detect possible aberration of the CCA and ICA. If aberrations are found, it is recommended that MRI examination of the brain be performed to determine whether infarction is present. If no infarction is found, preventive measures to avoid future stroke should be discussed with the patients.

## Authors

Koichi Tsunoda, Seiji Bito, Chong Tonghyo, Yohko Morita, Yoshiki Nakamura, Yoshinori Isobe, Yutaka Tokumaru, Takeshi Saitoh, Ryoh Suzuki, Nogusa Takahara, Tatsuki Fukushima, Yumiko Hosaka, Toshikazu Sumida, Fumihiro Takeguchi, Momoe Mizushina, Yasuharu Morimoto, Taku Yabuki, Yasuhiro Yamada, Kootaro Kakei, Junko Tanabe, Ryosuke Higuchi, Toshiyuki Ikeoka, Asako Onishi, Shuichi Kondo, Iwao Chishima, Hideo Morikawa, Kimitaka Kaga, Sumio Matsumoto (NHO, Tokyo Medical Center, Tokyo);

Tsutomu Numata (NHO Chiba Medical Center, Chiba);

Mamoru Doi (NHO Kamaishi Hospital, Kamaishi);

Haruka Nakahara (NHO Disaster Medical Center, Tokyo);

Hiroyuki Kuroda (NHO Kobe Medical Center, Kobe);

Masamitsu Senarita (NHO Mito Medical Center, Mito);

Toyota Ishii (NHO Sagamihara Hospital, Sagamihara);

Takafumi Takemura, Koichi Haba, Norihisa Ogata (NHO Kumamoto Medical Center, Kumatoto);

Tetsu Komuro, Fujinobu Tanaka (NHO Nagasaki Medical Center, Nagasaki);

Jun Tsuji (NHO Kyoto Medical Center, Kyoto);

Sho Hashimoto (NHO Sendai Medical Center, Sendai);

Tomoya Yamamoto (NHO Kyushu Medical Center, Fukuoka);

Shin-Ichi Ishimoto, Yoko Fujimaki (Syakaihoken Chuo Hospital, Tokyo);

Takao Yabe (Tokyo Metropolitan Hiroo Hospital, Tokyo);

Rie Naito (Metropolitan Neurological Hospital, Tokyo);

Toru Sasaki (Tokyo Kita Syakaihoken Hospital, Tokyo);

Shigeru Hirano (Kyoto University, Kyoto);

Kanako Masuda (Keio University, Tokyo);

Atsunobu Tsunoda (Tokyo Medical and Dental University, Tokyo);

Kenji Kondo, Sotaro Sekimoto, Tatsuya Yamasoba, Kenji Itoh (University of Tokyo, Tokyo).
